# Spontaneous Expulsion of a Sebaceous Cyst: A Case Report of a Rare Surgical Outcome

**DOI:** 10.7759/cureus.104116

**Published:** 2026-02-23

**Authors:** Maninder S Gill, Prabhjot Cheema

**Affiliations:** 1 General Surgery, Dr. Harvansh Singh Judge Institute of Dental Sciences and Hospital, Panjab University, Chandigarh, IND; 2 Anatomy, Dr. Harvansh Singh Judge Institute of Dental Sciences and Hospital, Panjab University, Chandigarh, IND

**Keywords:** epidermal cyst, inflamed, minimally invasive, sebaceous cyst, spontaneous expulsion

## Abstract

Sebaceous cysts are lesions commonly found on the skin. Although they can occasionally get infected and rarely have other complications, they seldom cause any serious problems to the patient. Traditional treatment is through an elliptical skin incision, which can be delayed if the cyst is infected. Other minimally invasive methods of excision/treatment have also been described, which result in less scarring, better cosmetic outcome, and low morbidity. The present case is about an unusual outcome that occurred during the management of a sebaceous cyst. The patient presented with an inflamed sebaceous cyst on the back that already burst; hence, initial antibiotic treatment followed by delayed excision of the cyst was planned. However, there was spontaneous expulsion of the cyst while dressing the lesion. Subsequently, wound care was done, and healing occurred in around 10 days. There was no evidence of recurrence at the one-year follow-up. This outcome is unexpected and rare, but was somewhat like what has been described in various minimal excision techniques, though occurring spontaneously. Hence, while managing such cases, expulsion of the cyst wall or its portions should be looked for, as that can change the outcome.

## Introduction

Sebaceous cysts or epidermal cysts are lined with true, stratified-squamous epithelium, derived from hair follicle infundibuli or traumatic inclusion. They can occur anywhere on the skin, are fixed to it, and usually have a central punctum. Treatment depends on the clinical state of the cyst. When inflamed or infected, they should be incised and drained initially and removed later once the inflammation and induration have subsided. It is important to excise the cyst in its entirety as failure to do so usually results in recurrence [1]. Although the standard method of treatment is through an elliptical incision, multiple other methods have also been described, but all emphasize the complete removal of its wall as failure to do so may result in recurrence [1,2].

## Case presentation

A 48-year-old, moderately built male patient presented with a history of a 1.5 cm spherical swelling on the left upper back region for around two years. For the last three to four days, the swelling was painful and had redness, and it had burst with the exudation of thick discharge one day ago. According to the patient, the pain was moderate, and the discharge was a few milliliters per day. There was no history of fever or any other symptom. There was no earlier episode of pain or discharge from the swelling, and it was asymptomatic till the current episode. The patient was hypertensive and non-diabetic.

On examination, there was a 1.5 cm swelling just medial to the medial border of the left scapula in the upper back. The swelling had redness, slightly raised temperature, and mild to moderate tenderness. There was an around 5 mm opening at the top of the swelling with thick yellowish discharge oozing out. The patient's total leucocyte count (TLC) was slightly elevated (11,400/cumm), but the rest of the complete blood count (CBC) and blood biochemistry were normal (Table 1).

**Table 1:** Hemogram and biochemistry laboratory results TLC: total leucocyte count; ESR: erythrocyte sedimentation rate; FBS: fasting blood count; S. creatinine: serum creatinine; H: high

Test	Reference range	Patient value
Hemoglobin (gm/dL)	13-16	13.2
TLC (cumm)	4,000-11,000	11,400 (H)
ESR (mm/hr)	0-20	18
FBS (mg/dL)	60-100	95
S. creatinine (mg/dL)	<1.1	0.95

A provisional diagnosis of an infected sebaceous cyst was kept. The treatment planned was antiseptic dressings, oral amoxicillin-clavulanate 625 mg three times a day, and oral paracetamol 650 mg SOS (si opus sit, meaning if needed or as required) for pain. Excision of the cyst was planned after four weeks.

While dressing, pressure was applied around the swelling to express as much discharge as possible. However, on pressing, the cyst started protruding through the opening, and with slight manipulation using artery forceps, the cyst was taken out. The cyst wall was found to be complete on gross examination. The cavity was irrigated with hydrogen peroxide (H2O2) and betadine solution, and dressing was done. The patient was sent home with advice for regular dressings and oral antibiotics as planned. Histopathological examination confirmed the diagnosis of a sebaceous cyst.

**Figure 1:**
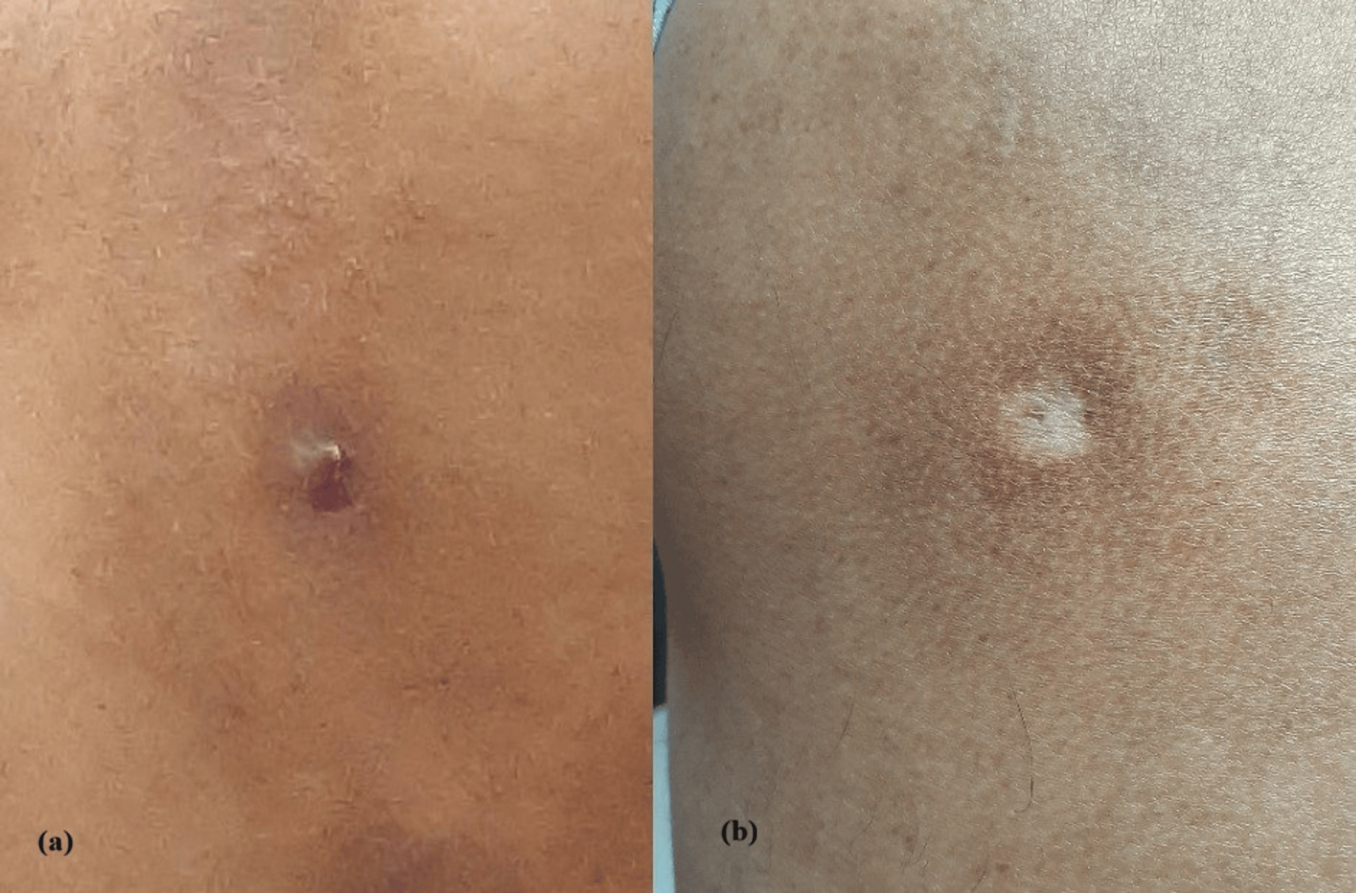
(a) Healthy wound with scab at day 8. (b) Completely healed wound at eight weeks with slight discoloration

The patient's wound healed in around 10 days, and there was no evidence of any lesion after eight weeks, except a slight discoloration of the skin (Figure 1).

The patient was asymptomatic at follow-up till one year.

## Discussion

Sebaceous cysts or epidermal cysts are very common and occur anywhere on the skin. They often arise from a ruptured pilosebaceous follicle associated with acne. The obstructed duct of the sebaceous gland in the hair follicle can result in a long, narrow channel opening in the surface comedo [2]. Although the standard surgical management is the elliptical excision technique, in which an elliptical strip of the skin is removed along with the cyst, minimal excision techniques have also been described with superior cosmetic results and less scarring [2-4]. These involve making a 2-3 mm incision, expressing the cyst contents through compression, and extracting the cyst wall through the incision [2]. Other methods described include excision using a suction system, in which a minimal incision is given and cyst contents are aspirated using a suction canula followed by the removal of the wall [5]. Removal of the contents with a laser punch, i.e., making a small hole with a laser and removing the contents through it, followed by the removal of the wall with a minimal excision about one month later [6] and nonsurgical management with recombinant hydrolytic enzymes [7] have also been described. Regarding the timing of excision for infected cysts, although the conventional approach is initial incision and drainage followed by interval excision, one-stage excision of an inflamed sebaceous cyst has also been described and is known to decrease the duration of antibiotic exposure and morbidity and is more economical [8].

In the current case, although delayed excision of the infected sebaceous cyst was planned, spontaneous expulsion of the cyst occurred while expressing the discharge through the already present opening. This happened as described in minimal excision techniques, though spontaneously. The possible reasons for this outcome could be the following: absence of any previous episode of infection, the cyst being unattached to the surrounding subcutaneous fatty tissue, and sloughing off of the punctum and adherent portion of the skin, thereby allowing for its spontaneous expulsion.

Hence, while managing infected sebaceous cysts that already burst, one should watch for the expulsion of any portions of the cyst wall. If it is possible to expel/extract the cyst wall, it can be tried to achieve the complete healing of the lesion without any recurrence.

## Conclusions

Sebaceous cyst is a common lesion on the skin requiring surgical management. Although the standard procedure of elliptical skin incision to excise the cyst is commonly done, other minimal excision techniques with better cosmetic outcomes and shorter treatment durations have also been described. However, lesions that are infected and already burst can show spontaneous expulsion of the whole or part of their wall, altering the management protocol. Hence, this possible outcome should be kept in mind while treating such lesions.
